# RNA interference-mediated knockdown of genes involved in sugar transport and metabolism disrupts psyllid *Bactericera cockerelli* (Order: Hemiptera) gut physiology and results in high mortality

**DOI:** 10.3389/finsc.2023.1283334

**Published:** 2023-10-18

**Authors:** Neda Arad, Jorge R. Paredes-Montero, Mosharrof Hossain Mondal, Nathaniel Ponvert, Judith K. Brown

**Affiliations:** ^1^ School of Plant Sciences, The University of Arizona, Tucson, AZ, United States; ^2^ Facultad de Ciencias de la Vida, Escuela Superior Politécnica del Litoral, ESPOL, Guayaquil, Guayas, Ecuador

**Keywords:** biopesticide, citrus greening, sugar metabolism, sugar transport, potato psyllid, RNAi

## Abstract

**Introduction:**

The causal agent of zebra chip of potato and vein-greening diseases of tomato is "*Candidatus* Liberibacter solanacearum" (CLso), a fastidious bacterium transmitted by the potato psyllid. In the absence of disease-resistant cultivars, disease management has relied on minimizing vector population size to reduce CLso transmission, which requires frequent insecticide applications. There is growing interest in the use of RNA interference (RNAi) technology to supplant traditional insecticides with biopesticides. This requires knowledge of genes essential for insect livelihood whose knockdown leads to significant mortality or other phenotypes. Such candidate genes can be evaluated by reverse genetics approaches to further corroborate predicted gene function.

**Methods:**

Here, five potato psyllid genes involved in sugar homeostasis in the potato psyllid gut, α-glucosidase1 (*AGLU1*), aquaporin2 (*AQP2*), facilitated trehalose transporter1 (*TRET1*), Trehalase1 (*TRE1*), and Trehalase2 (*TRE2*), were investigated as candidates for effective gene silencing. Potato psyllid dsRNAs were designed to optimize knockdown of gene targets. Third instar PoP nymphs were given a 48-hr ingestion-access period (IAP) on individual or groups of dsRNA in 20% sucrose. Mortality was recorded 0, 3, 5, 7, and 9 days post-IAP. Gene knockdown was analyzed 9 days post-IAP by quantitative real-time reverse-transcriptase polymerase chain reaction amplification.

**Results:**

The individual or stacked dsRNA combinations resulted in 20-60% and 20-40% knockdown, respectively, while subsequent psyllid mortality ranged from 20-40% to >60% for single and stacked dsRNA combinations, respectively. Reverse genetics analysis showed that simultaneous knockdown of the five selected candidate genes with predicted functions in pathways involved in sugar-homeostasis, metabolism, and -transport yielded the highest mortality, when compared with single or combinations of targets.

**Discussion:**

Results confirmed the functions afforded by psyllid gut genes responsible for osmotic homeostasis and sugar metabolism/transport are essential for livelihood, identifying them as potentially lucrative RNAi biopesticide targets and highlighted the translational relevance of targeting multiple nodes in a physiological pathway simultaneously.

## 1 Introduction

“*Candidatu*s (*Ca.*) Liberibacter solanacearum” (CLso) is an emergent bacterial pathogen of solanaceous crops (1, 2) that causes zebra chip disease of potato and vein-greening disease in tomato and pepper crops in North and Central America where it is endemic (3–5). The bacterium is transmitted by the potato or tomato psyllid (PoP) *Bactericera cockerelli* (Sulc) (Psylloidea: Triozidae) in a circulative, propagative manner and it persists in psyllids for the life of the vector (6). The Asian citrus psyllid (ACP) *Diaphorina citri* (Kuwayama) and relative of PoP, is the host and vector of “*Candiatus* Liberibacter asiaticus” (CLas), which is the causal agent of citrus greening disease, a threat to citrus production exacerbated by multiple, recent introductions of the ACP-CLas pathosystem to citrus-growing regions worldwide (7). After ingestion from an infected host plant “*Ca* Liberibacter” is taken into the alimentary canal during feeding, where the bacterium multiplies, accumulating in the filter chamber, midgut loop (anterior and posterior midgut), Malpighian tubules, and portions of the esophagus and hindgut of fourth immature instar and young (teneral) adults (4, 8–10). After exiting the gut to the hemolymph, “*Ca* Liberibacter” cells assume a rod-shaped form and translocate to the oral cavity and enter the salivary glands where they are acquired and transmitted to the host plant (11). Bacteriliferous potato psyllids are capable of transmitting CLso to a susceptible plant host with an ingestion-access period (IAP) of several hours (1, 12–15). Reducing psyllid population size to reduce the rate of “*Ca* Liberibacter” transmission has relied heavily on frequent applications of conventional insecticides (16), which has led to unsustainable production costs and ecological impacts (17). There is an increasing interest in the potential for dsRNA biopesticides for the management of phloem-feeding insect-transmitted pathogens such as ‘*Ca*. Liberibacter’ (18–21).

Psyllids are plant phloem feeders belonging to the Order: *Hemiptera* and suborder: *Homoptera*, a large group of phytophagous insects that specialize in feeding on the sucrose-rich photosynthates. The high concentration of sugar in plant phloem sap requires precise regulation of osmotic homeostasis during hemipteran feeding and regulation of sugar metabolism as the high concentration of sucrose in phloem photosynthate results in an osmotic pressure several times greater than that in psyllid tissue (22, 23). Consequently, the genes participating in sugar metabolism present promising targets for RNAi biopesticide design as exogenous perturbation in their expression via RNAi is expected to dysregulate the delicate balance of sugar metabolism and result in lethality.

Sucrose, the main sugar constituent in phloem photosynthate, is a disaccharide made up of glucose and fructose subunits connected by an α-1,2 linkage. Research conducted on the pea aphid Acyrthosiphon pisum (Harris), a hemipteran aphid species closely related to both ACP and PoP, has revealed that osmotic pressure regulation upon sucrose ingestion is primarily managed by two key complementary mechanisms. The mechanism responsible for breaking down sucrose into its constituent monosaccharides, glucose and fructose, involves the stoichiometric affinity between sucrose and a transglycosylating α-glucosidase (AGLU) (22, 24). To alleviate osmotic pressure in the insect gut the glucose cleavage products are converted into oligomers, lowering the molarity of sugar in the gut. This glycolytic cleavage reaction allows fructose to be rapidly assimilated from the gut, leaving the glucose subunit covalently linked to the α-glucosidase (25), which is either released from the enzyme in the presence of water, or when the sucrose concentration is high, is attacked by sucrose to form oligosaccharides (24). The oligosaccharides are excreted in the honeydew and the osmotic pressure is the gut is maintained at a physiologically-stable level (23). Glucose is also released as a monomer into the midgut lumen, absorbed, and mobilized to the fat body, where it serves as a precursor for trehalose synthesis (26). Trehalose is the most abundant sugar in insect hemolymph, (27, 28), where it contributes to multiple physiological processes, including stress protection, energy storage, and feeding behavior regulation (28–33). For trehalose to serve its essential functions or to be harvested as an energy store in distal tissues, it must be transported from the fat body throughout the insect. This is facilitated by a specific transporter, the facilitated trehalose transporter1 (TRET1), which allows freshly synthesized trehalose to enter the hemolymph (34–37). As energy or substrate is required in distal tissues, the enzyme trehalase (TRE) catalyzes the breakdown of trehalose into its constituent glucose monomers. Glucose from this process serves two vital roles for insect physiology: it enters the glycolytic cycle to produce energy, and is diverted into the glucosamine pathway for chitin production, which forms the structural framework of the insect body (28, 38).

Another important process for regulating osmotic homeostasis involves the rapid transfer of water from the hemolymph and distal gut tissues to the sap ingestion site, facilitated by the transmembrane water channel, aquaporin (39). Aquaporins are abundant in the gut of the pea aphid *Acyrthosiphon pisum* (Harris) and whitefly *Bemisia tabaci* (Genn.) and have been shown to regulate osmotic potential in cells where they are expressed (40, 41). In ACP, reduced expression of aquaporins was found to lead to increased glucose concentration in whole-psyllid homogenates, and resulted in nymphal mortality (39).

The objective of this study was to better understand hemipteran gut physiology, based on functional genomics analysis of a specific suite of genes shown to be expressed in the potato psyllid gut. Here, genes identified with predicted functions in several parallel processes and/or pathways essential for maintaining sugar homeostasis in the gut and body of the potato psyllid (‘Central type’) were selected for the reverse genetics-driven experiments. The genes selected for knockdown (downregulation) by ingestion of dsRNA were PoP α-glucosidase1 (*AGLU1*), facilitated trehalose transporter1 (*TRET1*), aquaporin2 (*AQP2*), and trehalase1 and 2 (*TRE1* and *TRE2*). To evaluate the physiological effect of RNAi-mediated knockdown of sugar metabolism/transport and osmotic homeostasis genes, potato psyllids were allowed an IAP of 48 after which mortality was recorded 0, 3, 5, 7, and 9 days, and gene knockdown was quantified by real-time reverse-transcription polymerase chain reaction amplification (RT-qPCR).

## 2 Materials and methods

### 2.1 Potato psyllid colonies

CLso-infected potato psyllid colonies were established with infected adults collected in a commercial greenhouse in Snowflake, AZ in 2004 (3). Psyllid colonies were reared on tomato plants (‘Roma’ *Solanum lycopersicum* (L.)) in an insect-proof cage maintained in an otherwise insect-free growth room (BugDorms, BioQuip Products, Rancho Dominguez, CA, USA) maintained at 23-25˚C with an L12:D12 photoperiod at The University of Arizona, Tucson, AZ. Potato psyllids were identified as the ‘Central’ type based on the mtCOI fragment that differentiates among haplotypes in the U.S. (42). The CLso haplotype was determined as ‘A’ based on a single nucleotide repeat (SSR) marker (240 base pairs) amplified by Lso-SSR-1F/1R primers (43) (data not shown). Psyllids were analyzed for the presence or absence of CLso 3-4 times per year, by PCR amplification with CLso-A-specific 16S rRNA gene primers, with cycling parameters as described (43–45).

### 2.2 Candidate gene target selection for potato psyllid

PoP candidate gene targets were selected for knockdown using a two-step process. First, genes of interest were queried against the genome of the Asian citrus psyllid (Diaci psyllid genome assembly version 1.1 https://www.ncbi.nlm.nih.gov/assembly/GCF_000475195.1) with BLASTn (Basic Local Alignment Search Tool), and subsequent hits were used to search the in-house maintained PoP transcriptome database (http://sohomoptera.arizona.edu/) (43–47). The minimum shared nucleotide identity was set at >98%, with confirmation by BLASTn analysis against the analogous ACP protein and those of selected related insects (homopterans), available in the UniProtKB/Swissprot database. Phylogenetic analysis was carried out at the amino acid sequence level with orthologs of closely related insect species, available in the GenBank database. To identify predicted functional orthologs the translated amino acid sequences were submitted to the KEGG (Kyoto Encyclopedia of Genes and Genomes) database using BlastKOALA (48).

The KEGG mapper pathway reconstruction tool (https://www.genome.jp/kegg/mapper.html) was implemented to assign PoP orthologs to a functional pathway. To identify potential off-target effects, each transcript sequence was searched against the PoP gut and salivary glands transcriptome (http://sohomoptera.arizona.edu/). Each sequence was divided into 21-nucleotide-long siRNA windows, shifting consecutively by one nucleotide, until the coding region scan was completed. Next, the predicted specificity and efficiency of siRNAs resulting from *in silico*-cleaved sequences was calculated using a custom in-house script (49). dsRNA primers (**Table 1**) and RT-qPCR TaqMan primer pairs and probe (**Table 2**) were designed using Geneious Prime software, version 8 (https://www.geneious.com/prime/) (50).

**Table 1:** Oligonucleotide forward (F) and reverse (R) primer pairs used for dsRNA synthesis of a fragment of each target gene, respectively, α-glucosidase1 (*AGLU1*), aquaporin2 (*AQP2*), facilitated trehalose transporter1 (*TRET1*), Trehalase1 (*TRE1*), and Trehalase2 (*TRE2*).

Target Gene Symbol	Sequence	Amplicon Size
*AGLU1*	F:5’ TAATACGACTCACTATAGGGTCACGGCTAAATACACACCTCTGG3’	245bp
	R: 5’ CTATAGTGAGTCGTATTATGCCGAACACAGGTTCGATGTCC3’	
*AQP2*	F: 5’TAATACGACTCACTATAGTCTGAGTTGTGTCAGCCTGCTGG3’	300bp
	R: 5’CTATAGTGAGTCGTATTATGCTTGTTGAGCGTGGTCATGCC3’	
*TRET1*	F: 5’TAATACGACTCACTATAGGAAAGCGCTGCAATGGCTAAGAGG3’	253bp
	R: 5’CTATAGTGAGTCGTATTAGTCAATGGTACTGCCAGCGTCC3’	
*TRE1*	F: 5’TAATACGACTCACTATAGAGAACTTTGAGGACGCCCAGGAG3’	258bp
	R: 5’CTATAGTGAGTCGTATTAAGTAGGTGTCCCAGTAGTACAGCTCC3’	
*TRE2*	F: 5’TAATACGACTCACTATAGATCCTGTCATCGTCCCAGGTGGTAG3’	219bp
	R: 5’CTATAGTGAGTCGTATTAACTTCACCATCGGAATGAGCAACGG3’	
*LUC*-T7	F: 5’TAATACGACTCACTATAGAGACCACTTCAACGAGTACGACTTCGTGC3’	328bp
	R:5’CTATAGTGAGTCGTATTAAGACCACGGTACATCAGCACCACCCGAAAGC3’	

**Table 2:** Forward (F) and reverse (R) primers and probe (P) sequences used for quantitative real-time reverse transcription polymerase chain reaction (RT-qPCR) amplification of potato psyllid transcripts, corresponding to the gut gene of interest, α-glucosidase1 (*AGLU1*), aquaporin2 (*AQP2*), facilitated trehalose transporter1 (*TRET1*), Trehalase1 (*TRE1*), and Trehalase2 (*TRE2*).

Target Gene Symbol	Sequence	Amplicon Size
*AGLU1*	F: 5’AAGACGGTCAACGGTCAAAG3’	111bp
	R: 5’GGCGAAAGCGTGGTAGTAAT3’	
	P: 5’TCTCATTCCAAGTCCATGCTGGCC3’	
*AQP2*	F: 5’TGCTGGTCCTGGTCATCTTC3’	122bp
	R: 5’TCCTGTGAAGTCAATGGCGG3’	
	P: 5’CGCTGGCTATCGGACTGACA3’	
*TRET1*	F: 5’CACAGTTTCCCTCGGATCAA3’	95bp
	R: 5’TACACCTCTCCTGCTCTTCCGTCC3’	
	P: 5’GGGAAACTCGACTGCCTATT3’	
*TRE1*	F: 5’GCAGTCGCCGCTACTACATA3’	116bp
	R: 5’CAGCGTCCCTATGTTGGTCT3’	
	P: 5’ACGCTCTCAACCCCCACTAC3’	
*TRE2*	F: 5’CTAGTCGCTGGTTTGTCC3’	226bp
	R: 5’CCTACTTCGTCATGCCAC3’	
	P: 5’TGGAATGCCGACTTGCTATCAC3’	
*RPL5*	F: 5’CTGGTGTGATTGCTGATGATATTG3’	109 bp
	R: 5’TCCACAGGTTTGGTGGATTT3’	
	P: 5’AGCTGACCCAACCCATGTCAAGAA3’	

The ribosomal gene 5 (RPL5) was selected as the reference gene for RT-qPCR amplification.

### 2.3 Molecular cloning of potato psyllid genes of interest

Amplification by PCR was carried out with REDTaq^®^ ReadyMix™ PCR reaction mix, supplemented with MgCl2 (Sigma-Aldrich^®^, USA), according to the manufacturer’s protocol. The PCR reactions contained 12.5µL of reaction mix, 2µL of PoP cDNA, 1µL of each primer, and 9.5µL nuclease-free water. Cycling parameters were an initial denaturation at 95°C for 2 min, followed by 30 cycles of denaturation at 95°C for 20 sec, annealing for 20 sec (TmCalculator; (https://tmcalculator.neb.com/), and extension at 72°C for 30 sec, with a final incubation at 72°C for 2 min. The expected size of amplicons was verified by agarose gel electrophoresis by loading 5μL of each amplicon on a 1.5% agarose gel containing GelRed™ (BIOGENERICA SRL, Mascalucia (CT), Italy). Electrophoresis was carried out in 1x Tris-Acetate-EDTA (TAE) buffer, pH 8.0 at 100volts for 45min. Amplicons were gel-purified using the Thermo Scientific GeneJET PCR Purification Kit, according to the manufacturer’s instructions.

The purified amplicons were cloned using the pGEM^R^-T Easy Plasmid Vector System I Promega (Cat. # A1360). The commercially-obtained non-target Luciferase amplicon (49) was ligated into psiCHECK™-2 plasmid (Promega Corporation, USA). Each reaction reagent contained 5µL of 2x Rapid ligation buffer, 1µL of pGEM^R^-T Easy vector (50ng), purified PCR product, 1µL of T4 DNA Ligase (3 Weiss units/µL), and up to 10µL deionized water. Reactions were incubated overnight at 4°C. The amount of purified PCR product was calculated with the NEBiocalculator (https://nebiocalculator.neb.com/#!/ligation). For bacterial transformation, 10µL of the ligation reaction was added to a tube containing DH5a competent cells and held on ice for 20 minutes. Transformation was carried out using the heat shock method. Bacterial pellets were collected by centrifugation and resuspended in 100µL SOC media and plated onto LB agar plates, supplemented with ampicillin and X-gal for blue-white color selection, overnight at 37°C. White colonies were screened by colony-PCR to identify plasmids that harbored the cloned gene of interest Amplicon size was estimated by agarose gel (1%) electrophoresis in Tris-acetate-EDTA buffer, pH 8.0. Colony-PCR products were subjected to confirmatory Sanger DNA sequencing at Eton Bioscience (www.etonbio.com). Sequences were edited using Geneious Prime ver.8 software and gene identification was confirmed by comparison with reference transcripts and/or exons in the ACP genome v.3. The plasmid vectors containing the clone gene fragments of interest were purified with the Fermentas/GeneJETTM Plasmid Miniprep Kit, according to the manufacturer’s instructions.

### 2.4 Synthesis of double-stranded RNA and psyllid ingestion-access period

Double-stranded RNA (dsRNA) was synthesized using the *in vitro* kit, MEGAscript™ T7 Transcription Kit (catalog # AM1334, ThermoFisher, Waltham, MA). The coding regions of target genes for dsRNA were amplified by PCR amplification using the REDTaq^R^ ReadyMix™ PCR Reaction Mix and T7 primers, and the amplicons were fractionated by agarose gel electrophoresis, as described above. The second strand of dsRNA was transcribed using a mixture of 10X reaction buffer, dNTPs, T7 enzyme, and 12.5μL of the PCR product. Reactions were incubated at 37°C for 13 hours. Post incubation, residual template DNA was removed using the TURBO DNA-free kit (Ambion, TX, USA), and dsRNA was precipitated by the addition of 30μL nuclease-free water and 30μL lithium chloride solution consisting of 7.5M lithium chloride, 50mM EDTA. Samples were incubated at -20°C for one hour, followed by centrifugation at 4°C for 15min at 16,000g. Pellets were washed with 1mL of 70% ethanol and the dsRNA was suspended in 20μL nuclease-free water. Quality and quantity of dsRNA were determined using the NanoDrop™ 2000/2000c spectrophotometer. The dsRNA was denatured at 95°C for 2.5min and allowed to cool slowly at room temperature. The integrity and expected size of each dsRNA were verified by agarose gel electrophoresis, as described.

Third instar PoP nymphs were collected from tomato plants, transferred to a plastic petri dish, and starved for six hours. Nymphs were transferred to a sucrose feeding chamber constructed from two plastic cups separated by parafilm stretched to create a sache holding 200µL of 20% (w:v) sucrose solution. A drop of green food coloring (McCormick Food Color) was added to sucrose to facilitate monitoring of ingestion-access, which was evident based on production of ‘green’ honeydew, as previously described (50).

A non-target dsRNA control consisting of Luciferase (328 bp) (AY535007.1:2532-4184) and a no-template water control were included in each experiment, as previously described (50). The non-target gene Luciferase-dsRNA concentration selected for experiments was equivalent to the total concentration of the target gene(s). Third instar PoP nymphs were given a 48-hour IAP on sucrose solution containing dsRNA at 100 ng/µL. Psyllids were transferred to tomato plants at the three-leaf stage and maintained until molting into teneral adults (50). Mortality was recorded on days 0, 3, 5, 7, and 9, post-IAP. Ten live teneral adults were collected and transferred to a 1.5mL microfuge tube containing two 20mm zirconium beads and stored at -80°C until RNA isolation. To confirm the stability of the dsRNA post-IAP, 5μL of sucrose solution from each feeding chamber was fractionated and visualized by agarose gel electrophoresis, as described.

### 2.5 Total RNA isolation, cDNA synthesis, and quantitative real-time reverse-transcriptase polymerase chain reaction for psyllids' genes

Post IAP, total nucleic acids were isolated from psyllids using the Tri Reagent kit (Sigma T9424). The specimens stored at -80°C were homogenized in TRI (1mL per ten insects) using the Storm 24 Bullet Homogenizer for 4 minutes. Samples were held at room temperature for 5 minutes, followed by centrifugation at 12,000g for 10 minutes at 4°C. The supernatant (800µL) was transferred to a fresh sterile tube and 200µL of chloroform was added. After shaking for 15 seconds, preparations were held at room temperature for 5 minutes and centrifuged at 12,000g for 15 minutes at 4°C. The aqueous phase was transferred to a clean, sterile tube to which an equal volume of isopropanol was added, with incubation at room temperature for 25 minutes, and centrifugation at 12,000g for 15 minutes at 4°C. The RNA pellet was washed by the addition of 1mL 75% ethanol, followed by centrifugation at 15,000g for 10 minutes at 4°C. The RNA pellet was air dried and rehydrated with 40µL of water. Genomic DNA was removed using the RNA Clean & Concentrator TM-5 kit (Zymo Research, Cat. # R1013) by the addition of 5µL DNaseI and 5µL DNA digestion buffer to 40µL of RNA, followed by incubation at room temperature for 15 minutes. The RNA binding buffer and 100% ethanol were added, mixed well, and transferred to a column, according to the manufacturer’s instructions. The quality and quantity of total RNA was assessed using the NanoDrop™ 2000/2000c Spectrophotometer.

The cDNA synthesis was carried out with 0.7μg RNA template for the High-Capacity cDNA Reverse Transcription Kit (Applied Biosystems 4368814, Carlsbad, CA 92008 USA). Each reaction consisted of 2µL of 10x RT buffer, 0.8µL of 25x dNTP Mix (100 mM), 2µL of 10x RT random primers, 1µL of MultiScribe™ reverse-transcriptase, 4.2µL of nuclease-free H2O, and 10µL of RNA. The PCR amplification was carried out with following cycling parameters: 10 minutes at 25°C, 120 minutes at 37°C, and 5 minutes at 85°C. The cDNA was diluted in 200μL of nuclease-free water.

The reaction efficiency of the TaqMan primer-probe combinations designed for RT-qPCR of psyllid transcripts was determined. A standard curve was established by RT-qPCR amplification of a 10-fold serial dilution of each cloned gene target respectively. The reaction efficiency was considered acceptable, if >90%. The purified plasmid harboring a cloned insert of each gene target was amplified by RT-qPCR with the respective TaqMan primers and probe combination. The reaction mixture contained 1μL of TaqMan primers-probe Master Mix (10μM), 25μL of the REDTaq^R^ ReadyMix™, 1μL purified plasmid (20ng/μL), and nuclease-free water to a total volume of 50μL. The amplicon size was estimated by agarose electrophoresis, as described above. The PCR product was purified and used as template in the ten-fold dilution series, from 0 to 10^-7^. The RT-qPCR reaction was carried out in the CFX96 Touch Deep Well Real-Time PCR System (Bio-Rad). The cycling conditions consisted of an initial step of 2 minutes at 50°C, an initial denaturation of 10 minutes at 95°C, followed by 40 cycles of 95°C for 15 seconds and 58°C for 60 seconds for TaqMan primer-probe annealing. The reaction mixture consisted of 10μL TaqPath™ RT-qPCR Master Mix, CG (2x) (Applied Biosystems™), 1μL TaqMan primer-probe Master Mix (20x), and 4μL template, and 5μL of nuclease-free water in a final reaction volume of 20μL. Three technical replicates and a no-template control (NTC) were carried out in a 96-well Microseal PCR plates (Bio-Rad1, Hercules, CA, USA). The Cq values and mean for each dilution were recorded. Using Excel, values were plotted to identify the slope of the best line through a minimum of three points. The primer efficiency was calculated using the following formula: (10^(-1/-slope)^) – 1.

Gene expression in mature PoP adults was quantified by RT-qPCR amplification, in triplicate, for each of three biological replicates, as described above. The relative gene expression of each PoP gene of interest was normalized based on expression of the *RPL5* Ribosomal protein large subunit 5 (RPL5) (GenBank KT185023). This reference gene was selected based the results of a previous study that showed RPL5 was stably expressed in potato psyllid (51). Fold-change was quantified using CFX Maestro RT-qPCR Analysis Software.

### 2.6 Statistical analysis

A minimum of three biological replicates were carried out for each experiment. Mortality rate associated with the water control was used as the baseline for adjusting mortality for the dsRNA treatments and non-target control, based on Abbott’s formula (52). This correction was made using the water control because characteristically, at least some mortality occurs due to handling the psyllids during collection and transfer steps. For each experiment, the corrected mortality was calculated for 0, 3, 5, 7, and 9 days post-IAP. The non-parametric Kruskal-Wallis test was used to identify statistically significant differences between treatment and control at each day post-IAP.

### 2.7 Rationale for CLso and RNAi experiments

The establishment of CLso-infected potato psyllid colonies served as a model for understanding the natural biology of psyllids in a controlled environment. We monitored the presence of CLso to ensure that our colonies were representative of psyllid abatement in practice, as CLso infection is prevalent in natural psyllid populations and incentivizes research into the development of biopesticides.

RNAi experiments were designed to explore gene silencing mechanisms in the potato psyllid - although RNAi experiments were not directly aimed at affecting the CLso status, they are inherently related to the overall framework of understanding how gene functions could be manipulated in psyllids, which are natural carriers of CLso.

## 3 Results

The objective of this study was to evaluate individual target genes, pairs of genes, and stacked gene combinations to identify the minimal number of dsRNAs required to decrease the survival of potato psyllids. Mortality rates were recorded for nine days post-IAP on dsRNA and the non-target and negative water control treatment, and the effects of exogenous dsRNA ingestion on PoP gene expression was analyzed 9 days post-IAP. An ideal target was considered one for which the minimal arbitrary threshold of 40% increased mortality could be achieved by dsRNA treatment, relative to the non-target control.

### 3.1 Mortality and knockdown post-treatment with three selected dsRNAs

**Figure 1:**
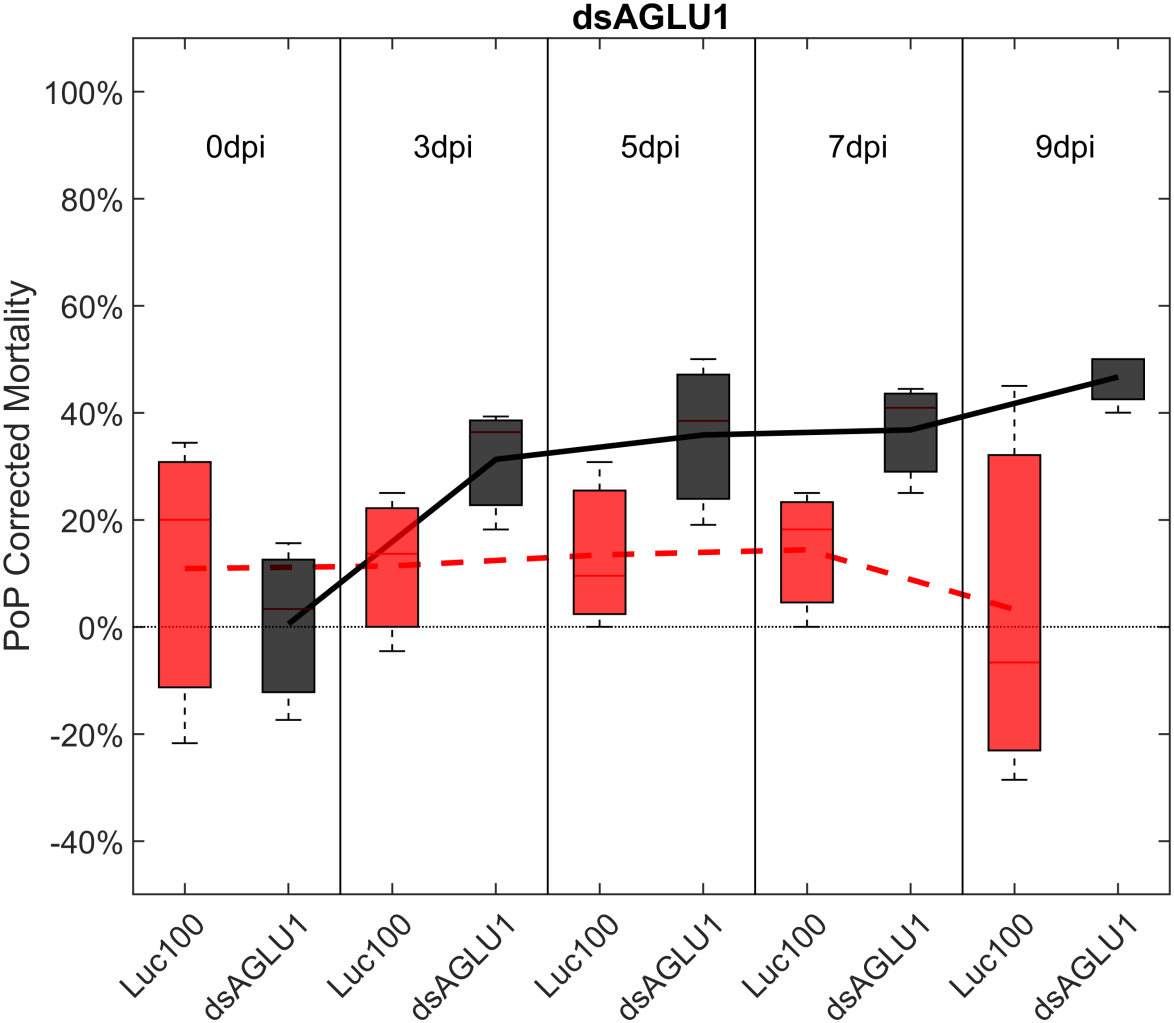
Mortality for PoP, on 0-, 3-, 5-, 7-, and 9dpi resulting from ingestion of a single dsRNA targeting α-glucosidase1 in 20% sucrose solution carried out for three replicates. ‘dpi’ indicates days post-ingestion. ‘Luc100’ refers to 100ng/μL of Luciferase dsRNA used as control. ‘dsAGLU1’ refers to dsRNA targeting α-glucosidase1, used as treatment. In each boxplot the center bar represents the median, and the top and bottom of the box represents the 25th and 75th percentiles of the data, respectively. Corresponding line graphs are drawn over the mean of the data for each dpi and were not used in the calculation of statistical significance. Black line indicates mortality over time for treatment, red line indicates mortality over time for Luciferase control.

For initial downstream comparisons with stacked dsRNA-mediated knockdown, three genes were selected for individual analysis. Results indicated that knockdown with dsAQP2 alone showed a 22.1% decrease in *AQP2* transcripts, relative to the Luciferase non-target control (**Figure S1**). PoP mortality post-IAP on dsAQP2 was 10% on day 0 and only two-fold higher by day 9 post-IAP (**Figure S2**). Second, knockdown with dsTRET1 showed 58.11% *TRET1* reduced gene expression (**Figure S1**), mortality was minimal, reaching only 20% by 9-days post-IAP (**Figure S3**). Even so, compared to the non-target control psyllids post-IAP of Luciferase-dsRNA, psyllids experienced significantly increased mortality by 9-days post-IAP. Effectively, the overall sensitivity of *TRET1* to gene silencing in PoP was negligible and far below the established 40% threshold for effective RNAi penetrance. This result may represent an example for which expression of an alternate isoform of the gene in the gut or elsewhere in the body may compensate for silencing of the targeted TRET1 gene. This is consistent with results reported in a previous study in which silencing of *AQP*2 was less effective than observed for other *AQP* orthologues (51). Third, for experiments in which PoP ingested dsRNA targeting α-glucosidase1 (dsAGLU1) alone, mortality was 19.38% on day 0 post-IAP and increased to 46.67% by day 9 post-IAP (**Figure 1**). Relative gene expression in PoP teneral adults 9-days post-IAP, indicated *AGLU1* silencing resulted in 59.15% knockdown, relative the Luciferase non-target control (**Figure S1**). This was the highest mortality among single targets observed for PoP post-IPA on dsAGLU1. While the magnitude of gene knockdown and mortality were lower in psyllids post-IAP on dsAQP2 than dsAGLU1, gene knockdown and mortality were directly correlated in the latter two experiments, but not for the *TRET1* isoform evaluated here.

### 3.2 Analysis of paired dsRNA ingestion by PoP

**Figure 2:**
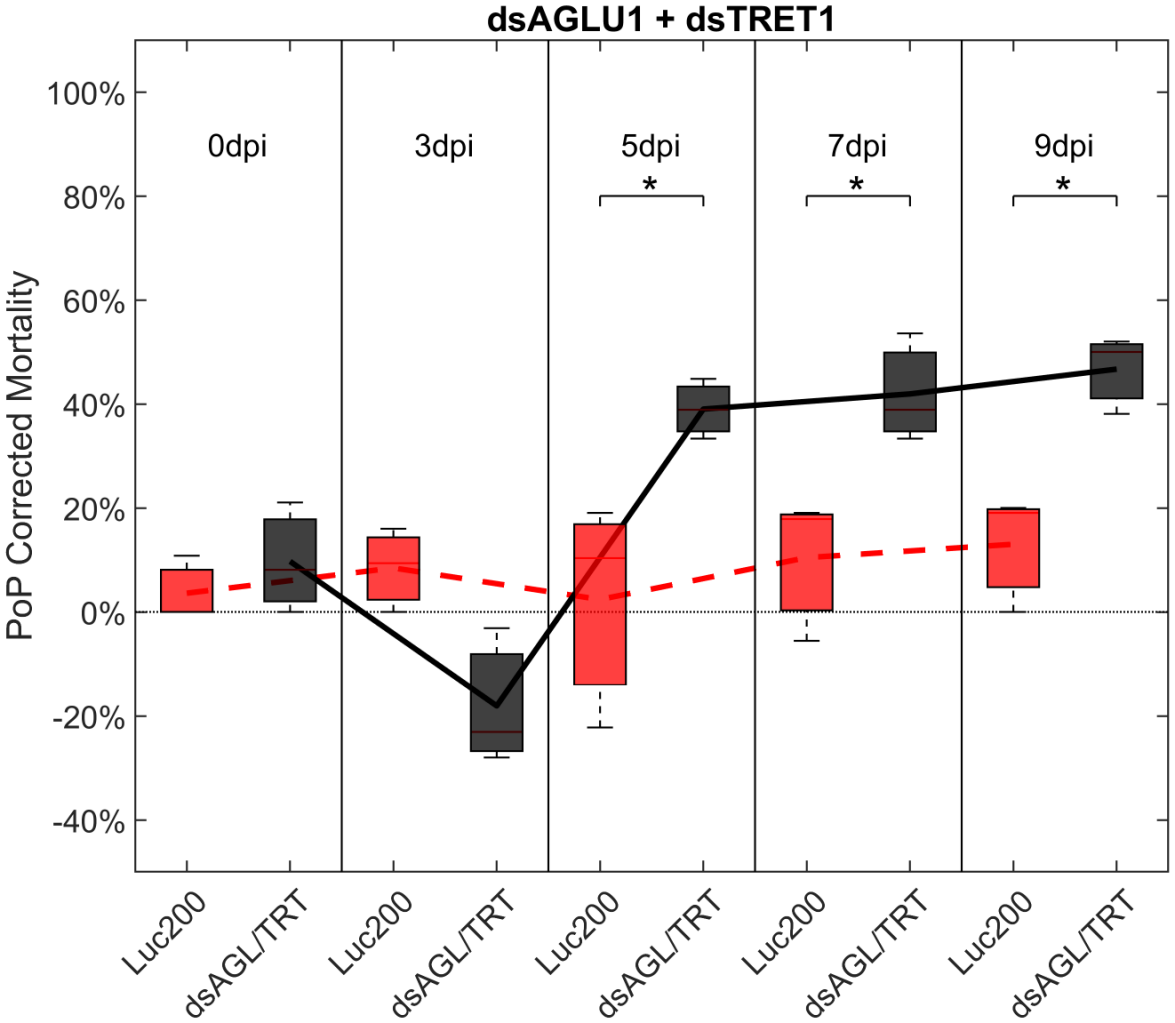
Mortality for PoP, on 0-, 3-, 5-, 7-, and 9dpi resulting from ingestion of two dsRNAs simultaneously, one targeting α-glucosidase1, and one targeting facilitated trehalose transporter1 in 20% sucrose solution carried out for three replicates. ‘dpi’ indicates days post-ingestion. ‘Luc200’ refers to 200ng/μL of Luciferase dsRNA used as control. ‘dsAGL/TRT’ refers to combination of dsRNAs targeting α-glucosidase1 and facilitated trehalose transporter1 simultaneously, used as treatment. In each boxplot the center bar represents the median, and the top and bottom of the box represents the 25th and 75th percentiles of the data, respectively. Corresponding line graphs are drawn over the mean of the data for each dpi and were not used in the calculation of statistical significance. Black line indicates mortality over time for treatment, red line indicates mortality over time for Luciferase control. Asterisks (*) indicate statistical significance at a p<0.05.

To determine if simultaneous knockdown of combinations of two of the five selected gut gene targets implicated in the same pathway and/or processes involved in sugar-homeostasis, -metabolism, and -transport would lead to additive and/or synergistic knockdown and/or mortality, two genes were targeted with exogenous dsRNA simultaneously. When psyllids were given a 48-hour IAP to dsAGLU1 and dsTRET1 simultaneously, knockdown of *AGLU1* and *TRET1* expression reached the arbitrary 40% threshold, at 41.22% and 31.52%, respectively (**Figure S4**). Mortality associated with *AGLU1* knockdown was 35.21% by day 3 post-IAP and increased to 46.70% by 9 days post-IAP (**Figure 1**), whereas mortality associated with silencing of *TRET1* remained around 20% for 3 to 9 days post-IAP (**Figure S3**). Compared to the non-target Luciferase-dsRNA treatment, significantly increased mortality was observed for 5, 7, and 9 days post-IAP when dsAGLU1 and dsTRET1 were fed simultaneously (**Figure 2**). Even though the mortality was slightly higher for dsAGLU1 co-delivered with dsTRET1, compared to either alone, only a slightly additive response was observed.

For the combined of dsAGLU1 and dsAQP2, knockdown of gene expression was 26.25% and 28.45% for *AGLU1* and *AQP2* expression, respectively (**Figure S4**), while the mortality rate was 23.32%, on 3 days post-IAP, but did not reach the 40% arbitrary threshold by 9 days post-IAP (**Figure S5**). However, compared to psyllids that ingested Luciferase dsRNA, a significant increase in mortality was observed at 5-, 7-, and 9-days post-IAP for this combination.

**Figure 3:**
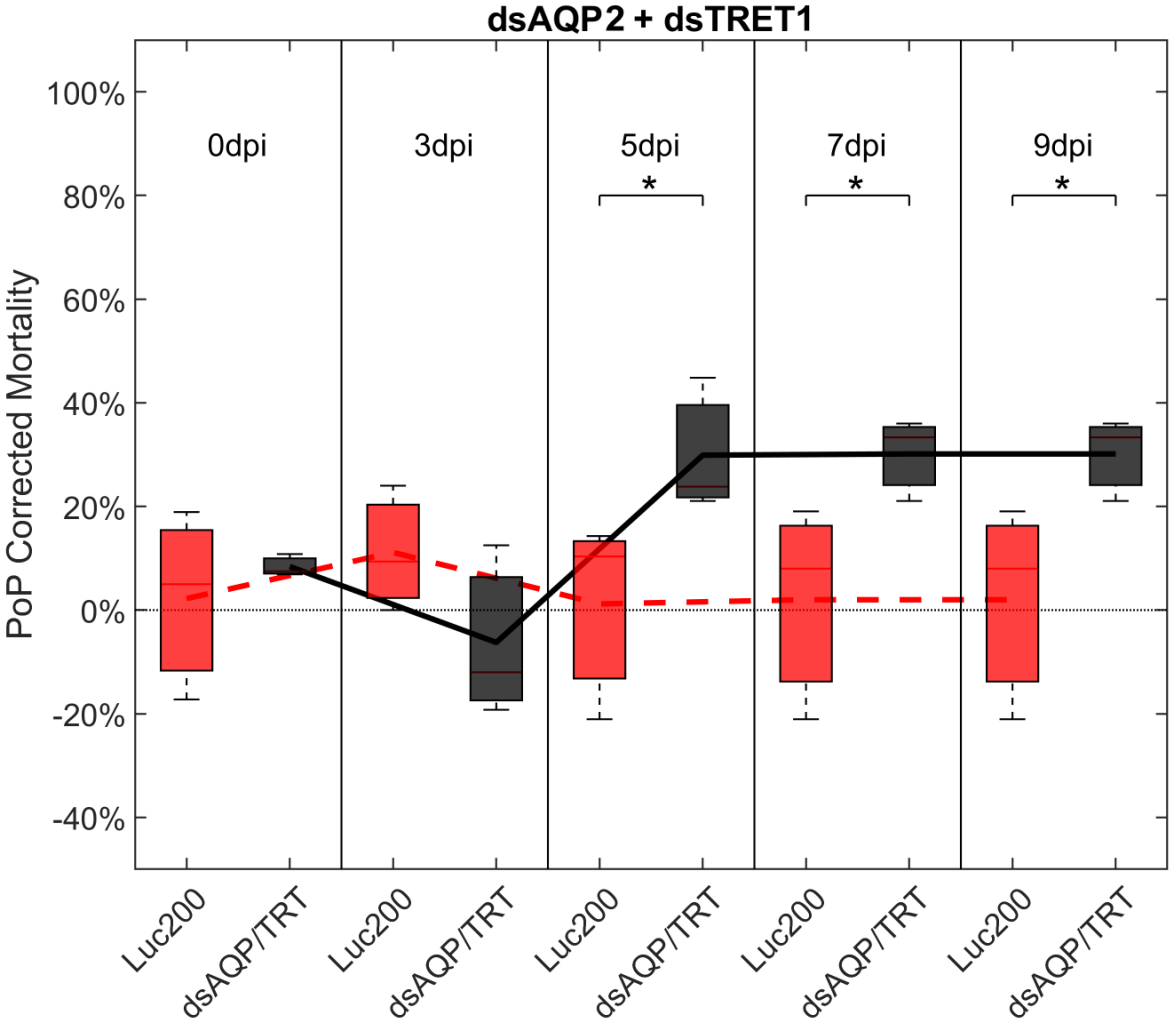
Mortality for PoP, on 0-, 3-, 5-, 7-, and 9dpi resulting from ingestion of two dsRNAs simultaneously, one targeting aquaporin2, and one targeting facilitated trehalose transporter1 in 20% sucrose solution carried out for three replicates. ‘dpi’ indicates days post-ingestion. ‘Luc200’ refers to 200ng/μL of Luciferase dsRNA used as control. ‘dsAQP/TRT’ refers to combination of dsRNAs targeting aquaporin2 and facilitated trehalose transporter1 simultaneously, used as treatment. In each boxplot the center bar represents the median, and the top and bottom of the box represents the 25th and 75th percentiles of the data, respectively. Corresponding line graphs are drawn over the mean of the data for each dpi and were not used in the calculation of statistical significance. Black line indicates mortality over time for treatment, red line indicates mortality over time for Luciferase control. Asterisks (*) indicate statistical significance at a p<0.05.

Ingestion-access to dsAQP2 and dsTRET1 simultaneously resulted in a 31.45% and 34.06% knockdown of *AQP2* and *TRET1*, respectively (**Figure S4**). Compared to Luciferase dsRNA, increased mortality was observed on 5-, 7-, and 9-days post-IAP. Initially, mortality was 23.39% at 3 days post-IAP but did not reach 40% by 9 days post-IAP, at 30%, indicating a moderate physiological response to knockdown. When compared to either dsRNA ingested alone, an additive effect was observed for the dsAQP2 and dsTRET1 combination where knockdown and mortality for two RNAs combined was equal to the sum of the effects of dsRNAs delivered separately (**Figures 1**, **3**, **S3**).

### 3.3 Analysis of stacked dsRNA ingestion by PoP

**Figure 4:**
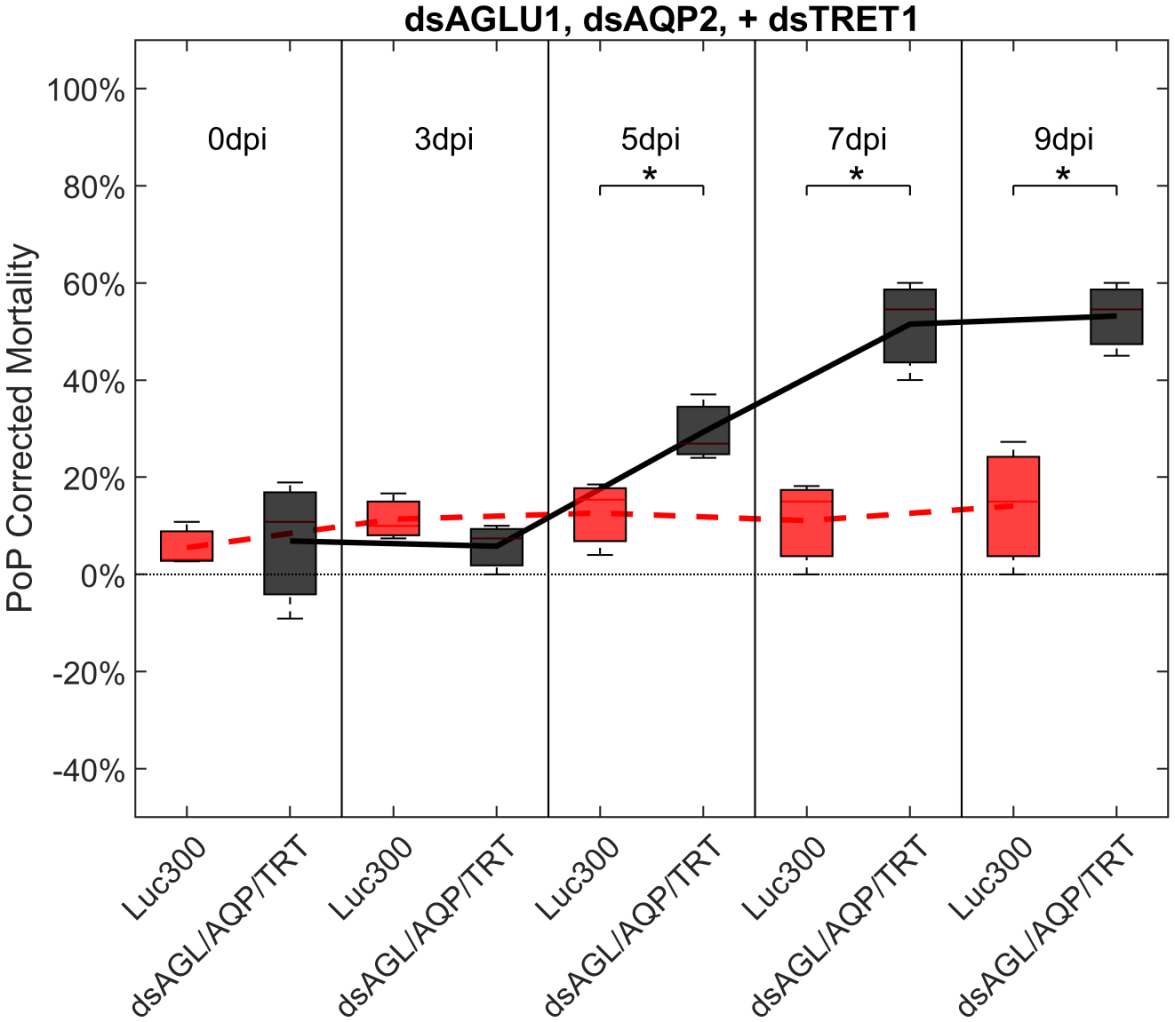
Mortality for PoP, on 0-, 3-, 5-, 7-, and 9dpi resulting from ingestion of three dsRNAs simultaneously, targeting α-glucosidase1, aquaporin2, and facilitated trehalose transporter1 in 20% sucrose solution carried out for three replicates. ‘dpi’ indicates days post-ingestion. ‘Luc300’ refers to 300ng/μL of Luciferase dsRNA used as control. ‘dsAGL/AQP/TRT’ refers to combination of dsRNAs targeting α-glucosidase1, aquaporin2 and facilitated trehalose transporter1 simultaneously, used as treatment. In each boxplot the center bar represents the median, and the top and bottom of the box represents the 25th and 75th percentiles of the data, respectively. Corresponding line graphs are drawn over the mean of the data for each dpi and were not used in the calculation of statistical significance. Black line indicates mortality over time for treatment, red line indicates mortality over time for Luciferase control. Asterisks (*) indicate statistical significance at a p<0.05.

Another objective of this study was to determine if the simultaneous suppression of multiple genes involved in sugar metabolism and transport would result in additive or synergistic effects on psyllid gut physiology. Ingestion by PoP of dsAGLU1, dsAQP2, and dsTRET1 resulted in gene knockdown of 45.28%, 40.40%, and 39.94% in the expression, respectively (**Figure S6**, top). This was associated with 21.36% mortality at 3 days post-IAP, which dramatically increased to 53.18% by 9 days post-IAP. The increased mortality at 5-, 7-, and 9-days post-IAP was significant when compared to the non-target Luciferase control and was well above the arbitrary 40% mortality threshold for RNAi penetrance (**Figure 4**).

**Figure 5:**
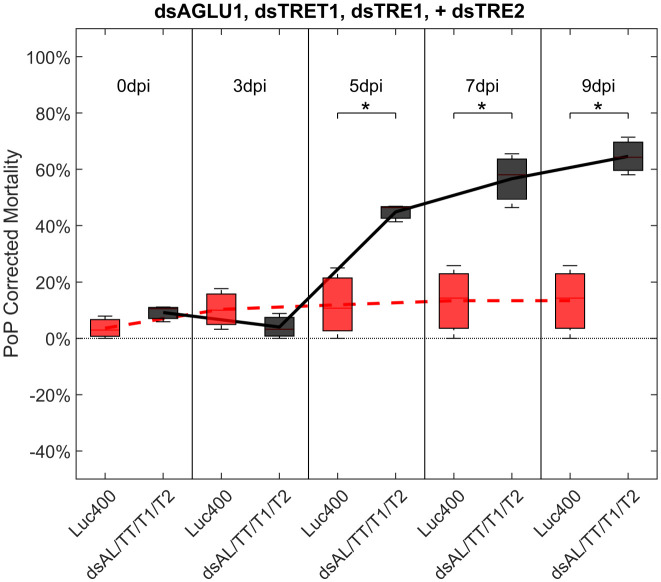
Mortality for PoP, on 0-, 3-, 5-, 7-, and 9dpi resulting from ingestion of four dsRNAs simultaneously, targeting α-glucosidase1, facilitated trehalose transporter1, trehalase1 and trehalase2 in 20% sucrose solution carried out for three replicates. ‘dpi’ indicates days post-ingestion. ‘Luc400’ refers to 400ng/μL of Luciferase dsRNA used as control. ‘dsAL/TT/T1/T2’ refers to combination of dsRNAs targeting α-glucosidase1, facilitated trehalose transporter1, trehalase1, and trehalase2 simultaneously, used as treatment. In each boxplot the center bar represents the median, and the top and bottom of the box represents the 25th and 75th percentiles of the data, respectively. Corresponding line graphs are drawn over the mean of the data for each dpi and were not used in the calculation of statistical significance. Black line indicates mortality over time for treatment, red line indicates mortality over time for Luciferase control. Asterisks (*) indicate statistical significance at a p<0.05.

To investigate additive effects, dsRNA targeting two trehalase genes were tested in two different experiments. First, dsTRE1, dsTRE2, dsAGLU1 and dsTRET1 were tested together. This combination resulted in the highest mortality at the earliest timepoint, of >40% at 5 days post-IAP. A significant increase in mortality was also observed compared to the Luciferase control at 5, 7, and 9 days post-IAP (**Figure 5**; **Figure S6**, middle).

**Figure 6:**
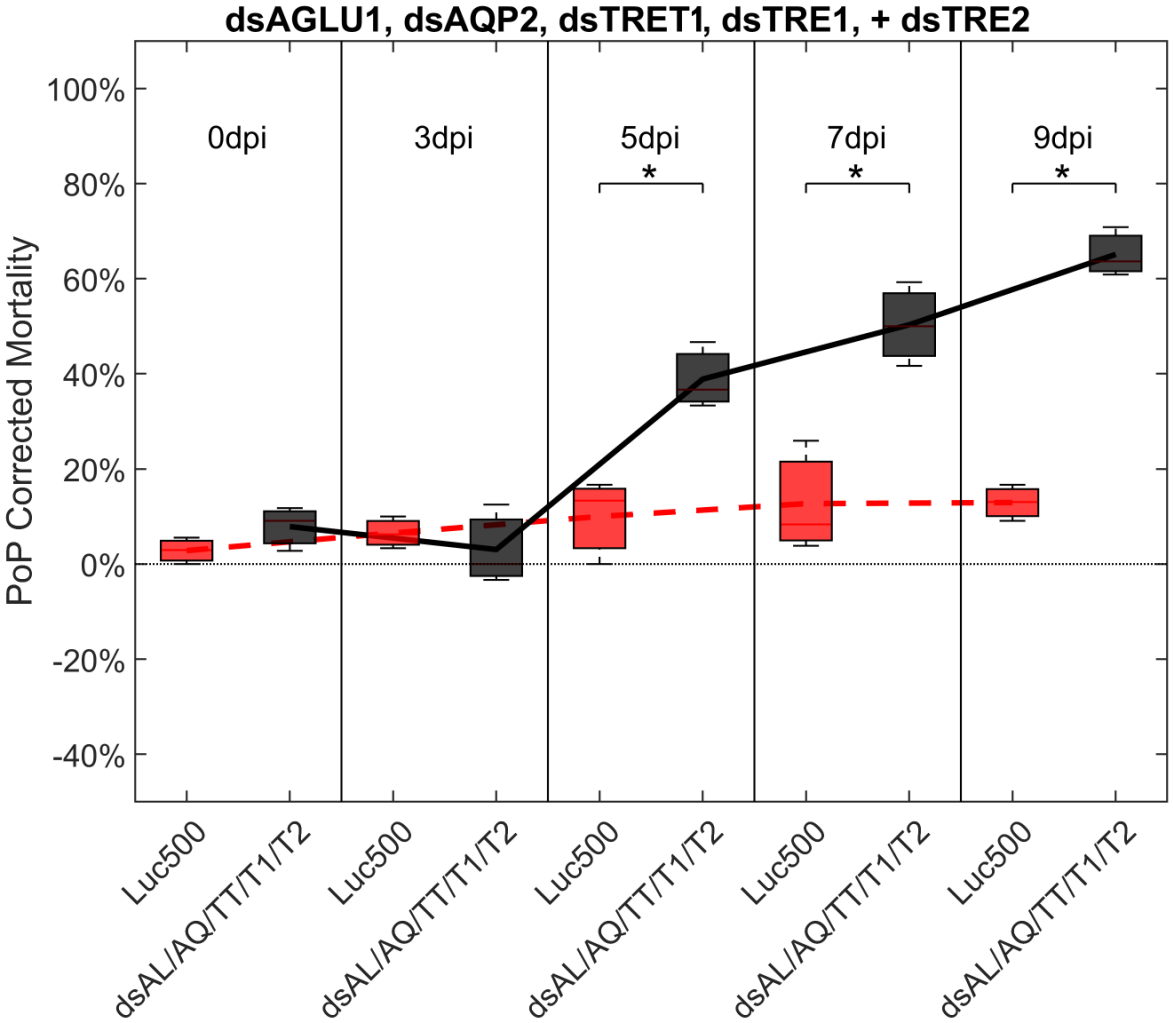
Mortality for PoP, on 0-, 3-, 5-, 7-, and 9dpi resulting from ingestion of five dsRNAs simultaneously, targeting α-glucosidase1, aquaporin2, facilitated trehalose transporter1, trehalase1, and trehalase2 in 20% sucrose solution carried out for three replicates. ‘dpi’ indicates days post-ingestion. ‘Luc500’ refers to 500ng/μL of Luciferase dsRNA used as control. ‘dsAL/AQ/TT/T1/T2’ refers to combination of dsRNAs targeting α-glucosidase1, aquaporin2, facilitated trehalose transporter1, trehalase1, and trehalase2 simultaneously, used as treatment. In each boxplot the center bar represents the median, and the top and bottom of the box represents the 25th and 75th percentiles of the data, respectively. Corresponding line graphs are drawn over the mean of the data for each dpi and were not used in the calculation of statistical significance. Black line indicates mortality over time for treatment, red line indicates mortality over time for Luciferase control. Asterisks (*) indicate statistical significance at a p<0.05.

Finally, psyllids were given a 48 hour IAP on dsTRE1, dsTRE2, dsAGLU1, dsAQP2, and dsTRET1. Mortality reached 65.11% by 9dpi (**Figure 6**). By comparison, mortality observed for the combination of dsAGLU1-dsAQP2-dsTRET1 (when two trehalases were omitted) was 53.18% by 9 days post-IAP (**Figure 4**). The high mortality associated with knockdown of all five gene targets showed an additive effect by 9 days post-IAP (**Figure S6**, bottom). Thus, targeting multiple essential nodes functioning in parallel in a single physiological pathway/process can result in enhanced mortality compared to silencing of one or even several essential genes. The knockdown values for the corresponding mortality rates can be found in **Table 3**.

**Table 3:** Potato psyllid gut gene targets analyzed for RNA interference experiments in which individual or all five dsRNAs combined were analyzed, corresponding to the gut gene of interest, α-glucosidase1 (*AGLU1*), aquaporin2 (*AQP2*), facilitated trehalose transporter1 (*TRET1*), Trehalase1 (*TRE1*), and Trehalase2 (*TRE2*), respectively.

Target Gene	Knockdown* (%)	
	4dsRNA	5dsRNA
*AGLU1*	47.17	41.09
*AQP2*	–	29.01
*TRET1*	51.16	34.16
*TRE1*	63.78	39.27
*TRE2*	53.23	20.16

*Relative gene expression level of PoP target genes normalized to the expression value of the RPL5 gene, and the changes were evaluated using CFX Maestro RT-qPCR analysis software (www.bio-rad.com). The student’s t-test significant groups (p-value <0.0.5) are indicated by the asterisk (*) when the experimental group was compared to the control group.

## 4 Discussion

RNA interference (RNAi), a defense mechanism that cells use to protect themselves from viruses by targeting and degrading RNA, has significant potential for controlling insect pests and vectors of plant pathogens, including “*Ca* Liberibacter”. In this study, we sought to marry two important aspects of potato psyllid biology: their status as CLso vectors and the potential for targeted gene silencing via RNAi. Although our RNAi experiments did not directly address CLso infection, understanding the functional genomics of potato psyllid will offer insights into the vector-pathogen biology of this pathosystem and eventually lead to novel management strategies for controlling the spread of CLso. This study demonstrated drastic RNAi effects in PoP following the ingestion of dsRNA from a sucrose diet. Several target genes considered in this study have been previously examined for individual downregulation in psyllids and other species (39, 53–55) due to their crucial roles in managing sugar metabolism and osmotic homeostasis (22, 24, 29, 56, 57). In the case of ACP, the vector of CLas, the causative agent of Citrus Greening Disease, downregulation of aquaporin led to increased nymph mortality and alterations in sugar homeostasis in adult psyllids (39). Moreover, it has been found that infection of ACP by CLas results in a significant shift in ACP trehalose metabolism (14). However, the above studies only targeted individual genes and, for some of the tested targets, reported low mortality corresponding to treatment, suggesting that RNAi-based genetic repression of single targets may have a limited effect when compared to repression of multiple targets, as reported here. The data collected in this study showed that feeding combinations of dsRNAs simultaneously led to higher mortality rates than single targets, implying that targeting an entire physiological system can result in more effective pest management, especially a system closely tied to psyllid livelihood.

**Figure 7:**
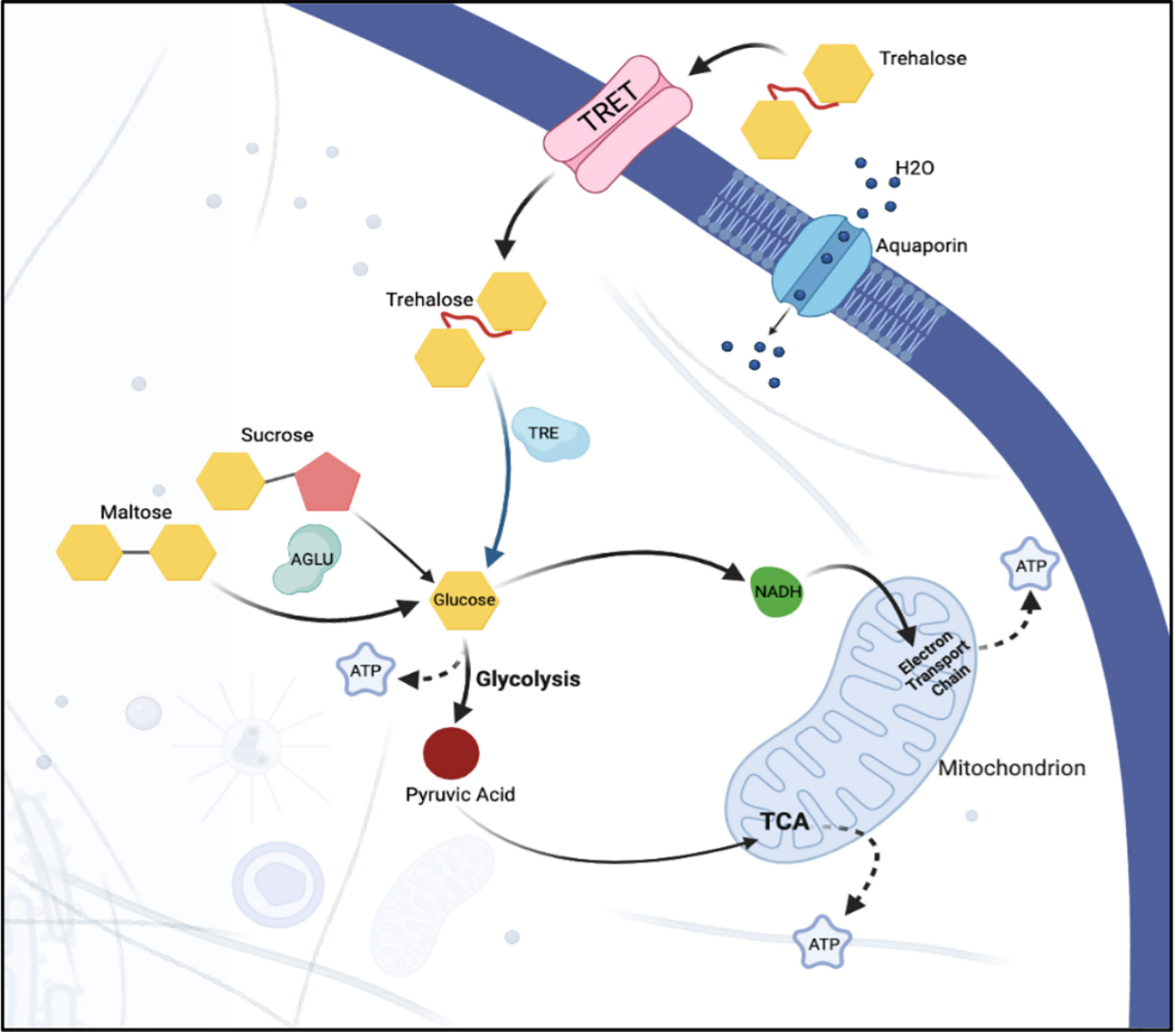
Sugar metabolism and transport in a hypothetical epithelial cell. Sugar, in the form of trehalose, is transported into the cell via facilitated trehalose transporter TRET. Once inside the cell, the enzyme trehalase hydrolyzes the trehalose into two glucose molecules. α-glucosidase breaks down sucrose into its constituent monosaccharides, facilitating its use in various cellular metabolic processes. Osmotic homeostasis within the cell is regulated via aquaporins that facilitate the passive transport of water. Together, these components ensure the efficient uptake and utilization of sugar and the maintenance of water balance within the epithelial cells of the psyllid.

While each of the target genes analyzed in this study were selected based on their predicted and/or known functions at important regulatory nodes of sugar homeostasis and osmoregulation (**Figure 7**), the extent of silencing of the different genes was variable. For example, *AGLU1* knockdown led to significant mortality, suggesting that it might play a more crucial role in the insect’s survival. In contrast, *TRET1* silencing resulted in minimal mortality, despite substantial knockdown, indicating the existence of possible compensatory mechanisms or redundant pathways. The results also suggest that targeting *AQP*2, both individually and in combination with other targets, yields only a slight mortality response. While *AQP2* appears to be less integral to psyllid sugar metabolism, the observed effects may have been due to yet-unknown interactions between AQP2 and other genes, or potentially, to compensatory expression of AQP orthologues.

This study shows that silencing multiple genes that perform independent, parallel functions in a single physiological pathway simultaneously can lead to additive or synergistic effects. Simultaneous knockdown of multiple genes at once has the potential to increase mortality, as was observed post-IAP of *AGLU1*, *AQP2*, and *TRET1* combined, compared to knockdown of the genes individually. By silencing multiple targets functioning within one physiological pathways, compared to the traditional single gene target approach, dsRNA biopesticides can be developed with greater efficacy against phloem-feeding insects such as the potato psyllid that may result in more effective pest control outcomes. Direct analysis of the RNAi products (small RNAs) in the psyllid gut and whole body in dsRNA-treated psyllids is expected to lead to a deeper understanding of the omics-effects of disrupting sugar homeostasis. This pre-screening, which was carried out using the ‘fast-track’ potato psyllid dsRNA pipeline, has facilitated the identification of *AGLU1*, *TRET1*, and *TRE1*/*2* as superior targets over *AQP*2 and *TRET1*, for advancement to screening in the ACP–CLas system, a major goal of the overall project.

In summary, key PoP gene targets have been identified that can be advanced for efficacy testing in ACP. Further, a foundation has been established for the development of biopesticides designed to knockdown multiple genes that function in the same pathway simultaneously, compared to traditional single gene target approaches. Finally, the research sets a new precedent for enhancing RNAi efficacy in insects that exhibit low RNAi penetrance by adopting a ‘dsRNA stacking strategy’ that undermines multiple inter-related physiological functions simultaneously. The strategy serves as a stepping-off point for dsRNA biopesticide screening in other phloem-feeding insects pests and vectors of economic importance.

## Data availability statement

The datasets presented in this study can be found in online repositories. The names of the repository/repositories and accession number(s) can be found in the article/**Supplementary Material**.

## Ethics statement

The manuscript presents research on animals that do not require ethical approval for their study.

## Author contributions

NA: Conceptualization, Data curation, Formal Analysis, Investigation, Methodology, Writing – original draft, Writing – review & editing, Validation, Visualization. JP: Methodology, Writing – review & editing. MM: Methodology, Writing – review & editing, Conceptualization. NP: Data curation, Visualization, Writing – review & editing, Formal Analysis. JB: Conceptualization, Funding acquisition, Methodology, Project administration, Resources, Supervision, Writing – review & editing.
