# Supplementary figures and images for: RNA interference-mediated knockdown of genes involved in sugar transport and metabolism disrupts psyllid Bactericera cockerelli (Order: Hemiptera) gut physiology and results in high mortality
Source: Front Insect Sci. 2023 Oct 18;3:1283334. doi: 10.3389/finsc.2023.1283334 (PMC10926392; doi:10.3389/finsc.2023.1283334)

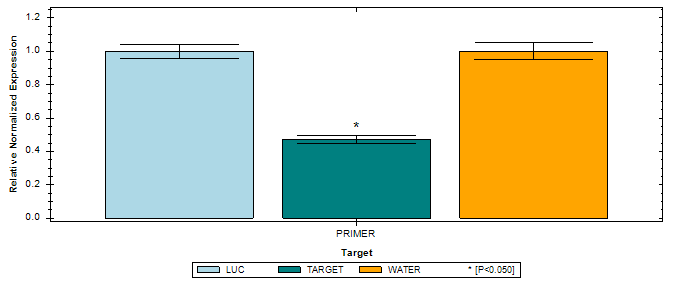

Supplement: Supplementary file 1 [file DataSheet_1.zip › qPCR_data_files/AGLU1/4ds_AGLU1_2022-03-03 16-50-17_CYCLER2.png]

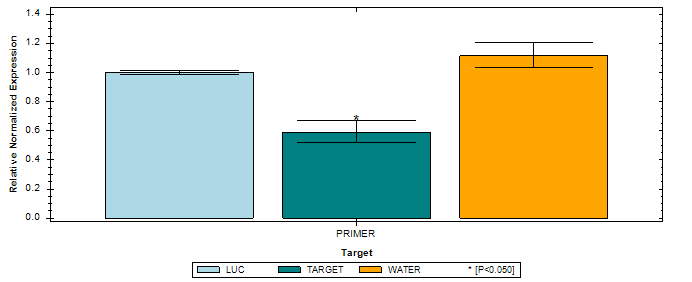

Supplement: Supplementary file 1 [file DataSheet_1.zip › qPCR_data_files/AGLU1/5ds_AGLU1_2022-03-22 13-54-11_CYCLER2.png]

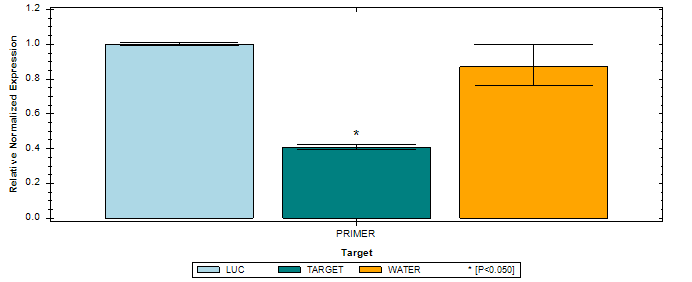

Supplement: Supplementary file 1 [file DataSheet_1.zip › qPCR_data_files/AGLU1/Aglu1_dsAglu1_2022-02-26 15-42-22_CYCLER3.png]

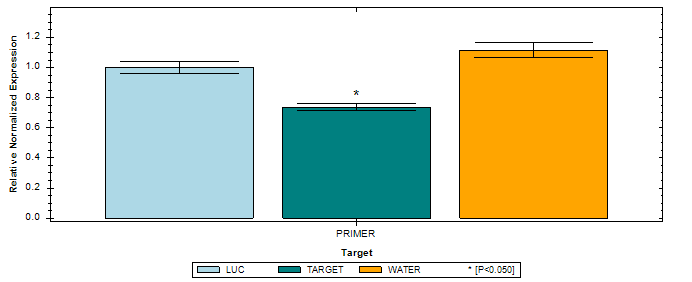

Supplement: Supplementary file 1 [file DataSheet_1.zip › qPCR_data_files/AGLU1/Aglu1_dsAglu1dsAQP2_2022-02-23 22-54-06_CYCLER1.png]

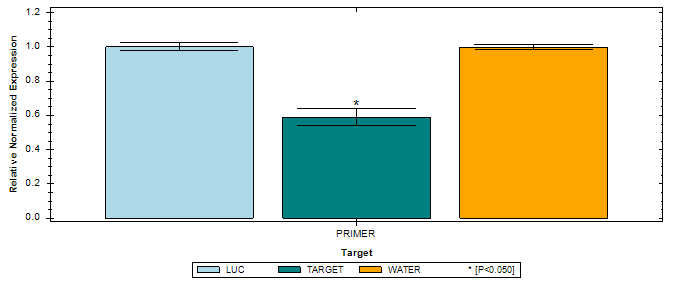

Supplement: Supplementary file 1 [file DataSheet_1.zip › qPCR_data_files/AGLU1/Aglu1_dsAglu1dsTret1_2022-02-23 21-48-49_CYCLER2.png]

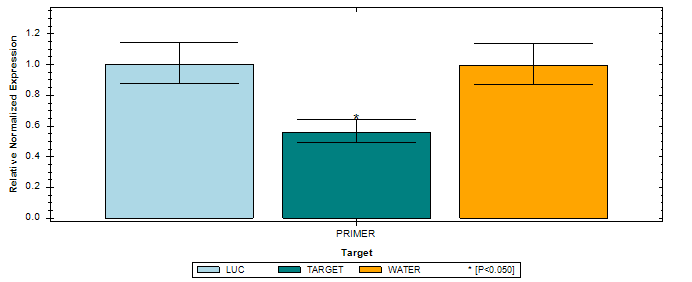

Supplement: Supplementary file 1 [file DataSheet_1.zip › qPCR_data_files/AGLU1/Aglu1_dsSTACKED_Rep1,2,3_2022-02-22 20-34-08_CYCLER2.png]

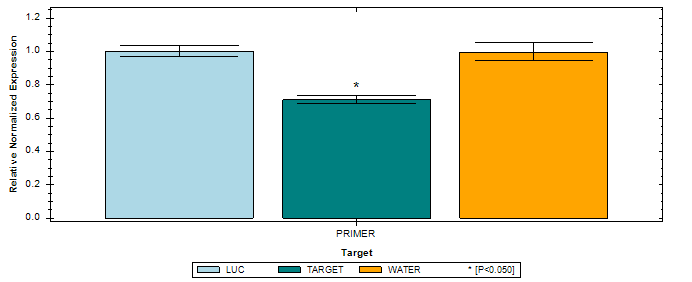

Supplement: Supplementary file 1 [file DataSheet_1.zip › qPCR_data_files/AQP2/5ds_AQP2_2022-03-22 16-56-41_CYCLER2.png]

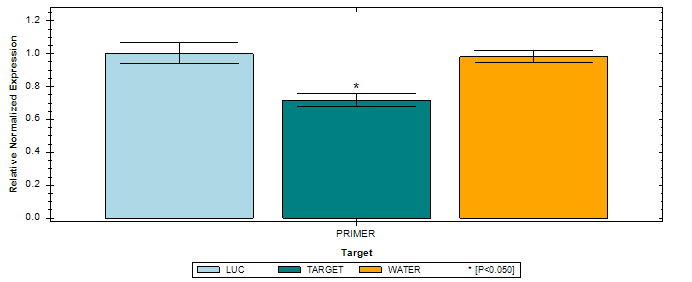

Supplement: Supplementary file 1 [file DataSheet_1.zip › qPCR_data_files/AQP2/AQP2_dsAglu1dsAQP2_2022-02-23 22-52-32_CYCLER3.png]

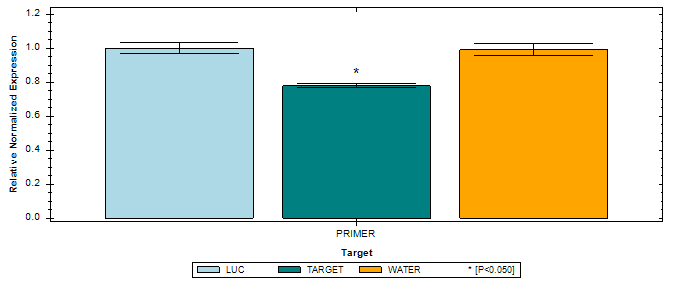

Supplement: Supplementary file 1 [file DataSheet_1.zip › qPCR_data_files/AQP2/AQP2_dsAQP2_2022-02-26 12-31-11_CYCLER1.png]

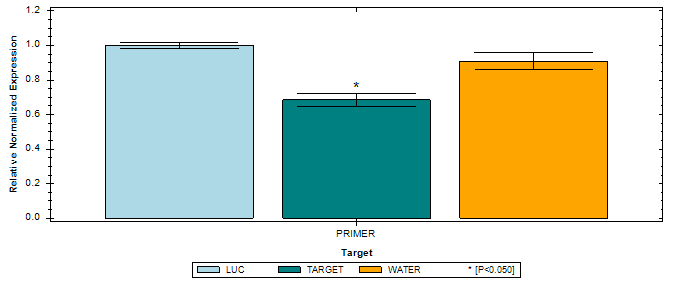

Supplement: Supplementary file 1 [file DataSheet_1.zip › qPCR_data_files/AQP2/AQP2_dsAQP2dsTret1_2022-02-23 20-30-59_CYCLER3.png]

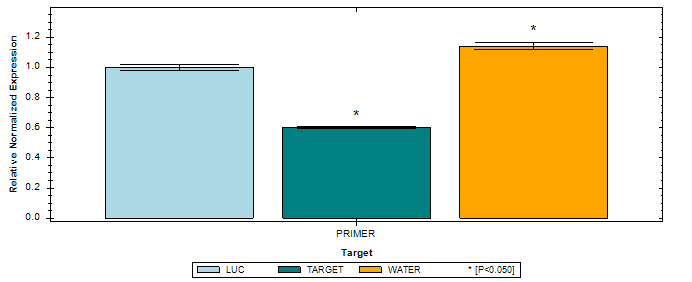

Supplement: Supplementary file 1 [file DataSheet_1.zip › qPCR_data_files/AQP2/AQP2_dsSTACKED_Rep1,2,3_2022-02-22 21-45-39_CYCLER1.png]

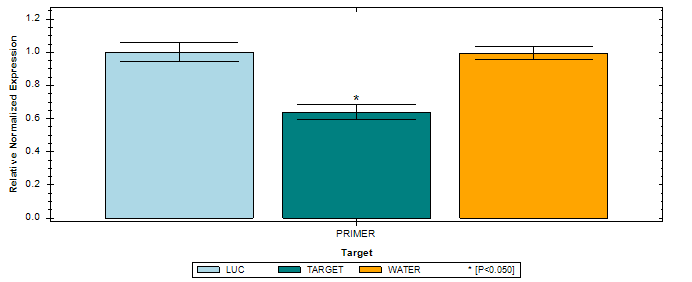

Supplement: Supplementary file 1 [file DataSheet_1.zip › qPCR_data_files/TRE1/4ds_TRE1_2022-03-03 18-53-21_CYCLER2.png]

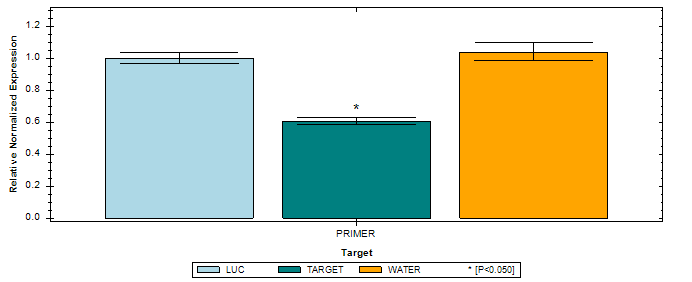

Supplement: Supplementary file 1 [file DataSheet_1.zip › qPCR_data_files/TRE1/5ds_TRE1_2022-03-21 13-17-33_CYCLER2.png]

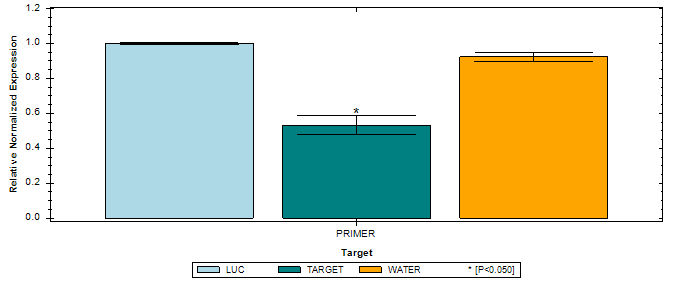

Supplement: Supplementary file 1 [file DataSheet_1.zip › qPCR_data_files/TRE2/4ds_TRE2_2022-03-03 20-28-24_CYCLER2.png]

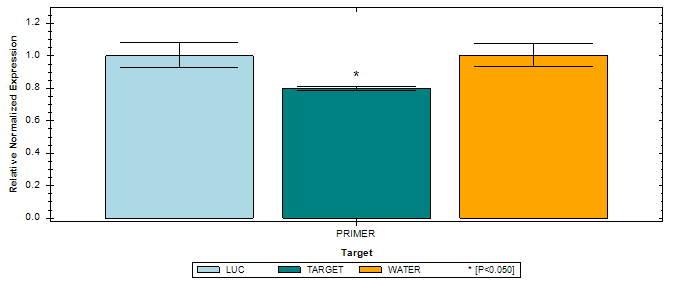

Supplement: Supplementary file 1 [file DataSheet_1.zip › qPCR_data_files/TRE2/5ds_TRE2_2022-03-23 14-54-11_CYCLER2.png]

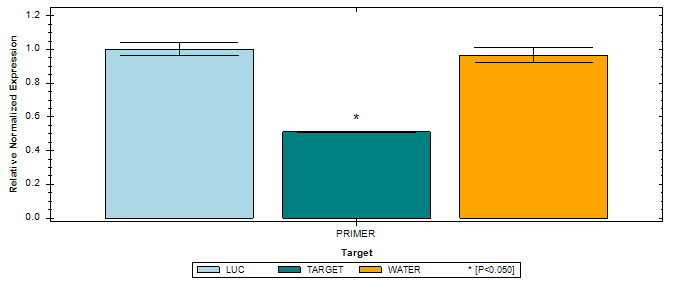

Supplement: Supplementary file 1 [file DataSheet_1.zip › qPCR_data_files/TRET1/4ds_TRET1_2022-03-03 14-50-44_CYCLER2.png]

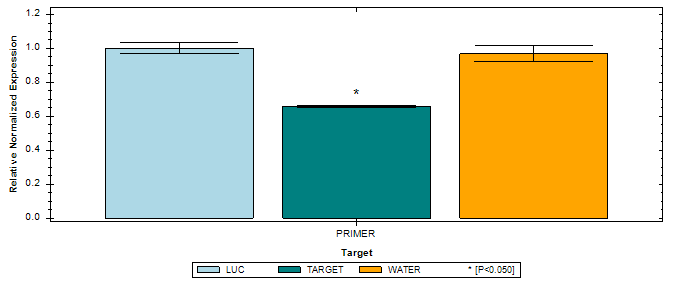

Supplement: Supplementary file 1 [file DataSheet_1.zip › qPCR_data_files/TRET1/5ds_TRET1_2022-03-18 15-30-00_CYCLER2.png]

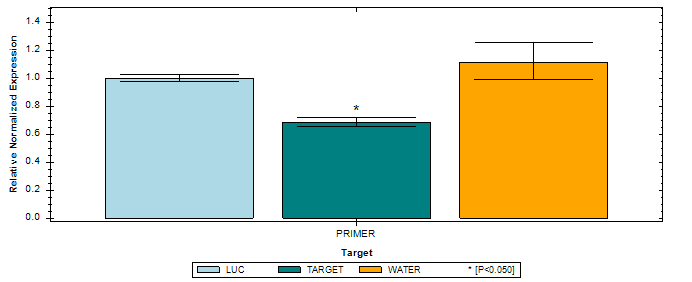

Supplement: Supplementary file 1 [file DataSheet_1.zip › qPCR_data_files/TRET1/Tret1_dsAglu1dsTret1_2022-02-23 21-01-05_CYCLER1.png]

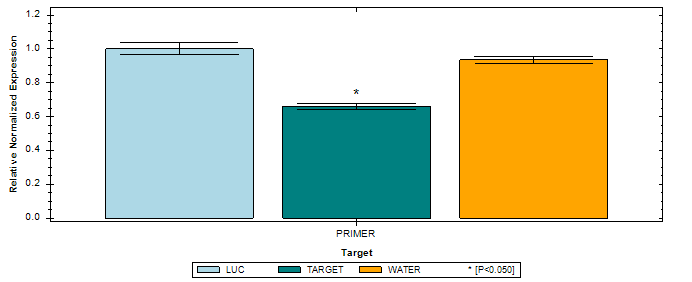

Supplement: Supplementary file 1 [file DataSheet_1.zip › qPCR_data_files/TRET1/Tret1_dsAQP2dsTret1_2022-02-23 20-12-11_CYCLER2.png]

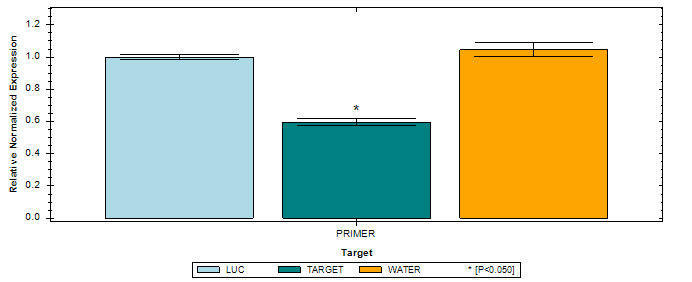

Supplement: Supplementary file 1 [file DataSheet_1.zip › qPCR_data_files/TRET1/Tret1_dsSTACKED_Rep1,2,3_2022-02-22 21-13-43_CYCLER3.png]

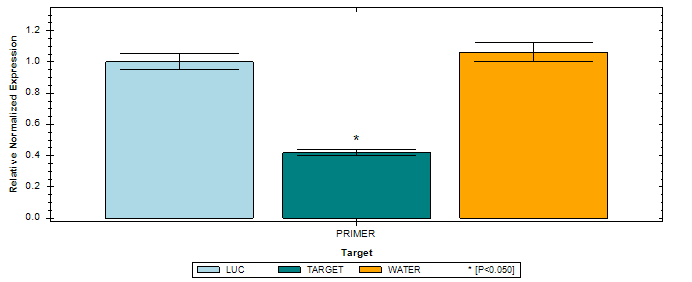

Supplement: Supplementary file 1 [file DataSheet_1.zip › qPCR_data_files/TRET1/Tret1_dsTret1_2022-02-26 15-15-26_CYCLER2.png]

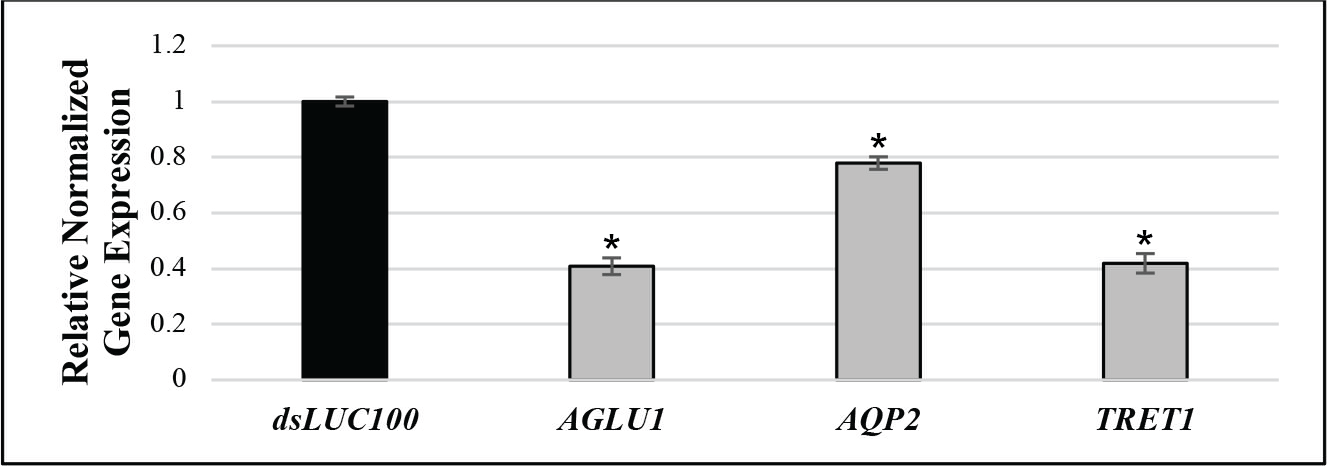

Supplement: Supplementary file 3 [file Image_1.tif]

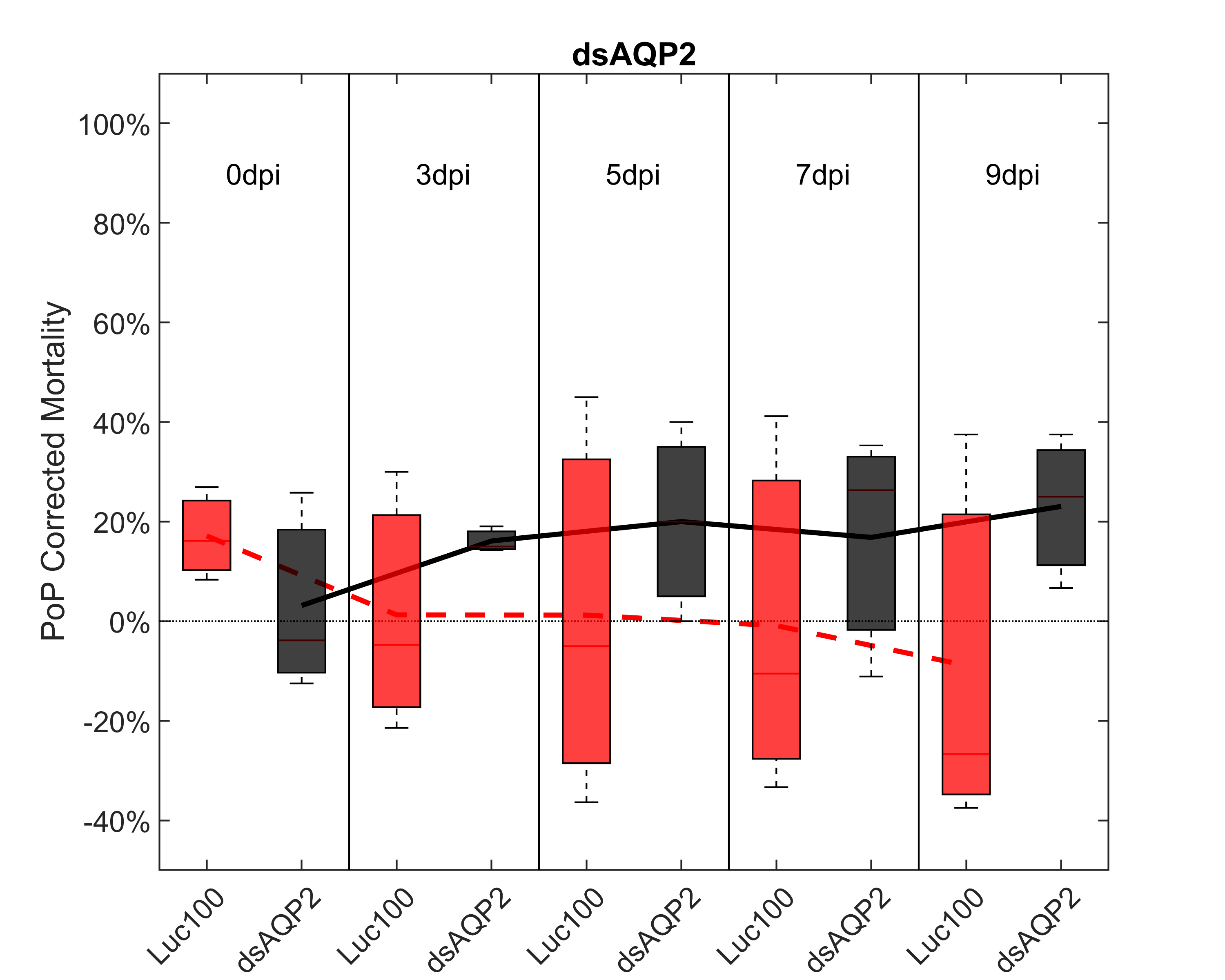

Supplement: Supplementary file 4 [file Image_2.tif]

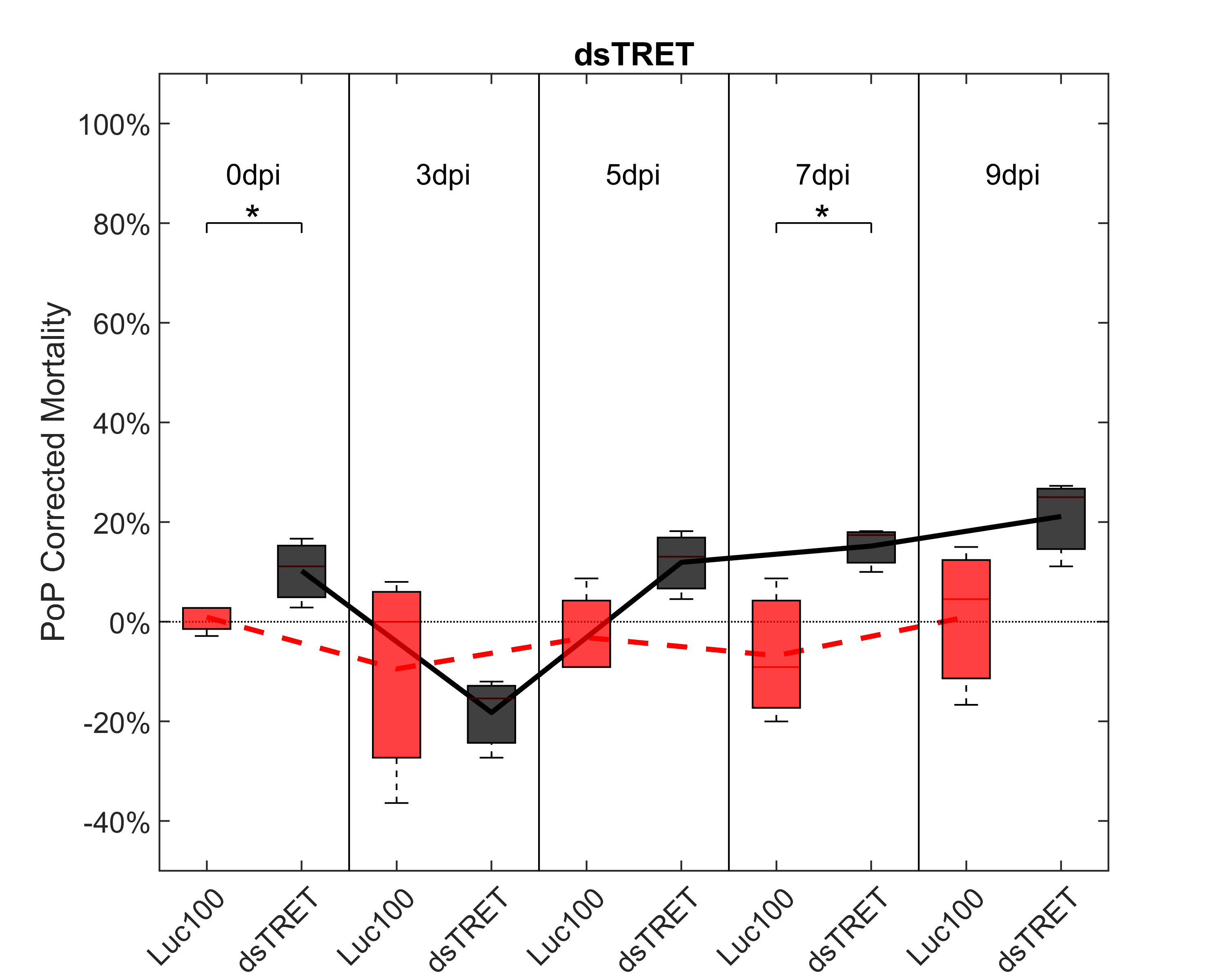

Supplement: Supplementary file 5 [file Image_3.tif]

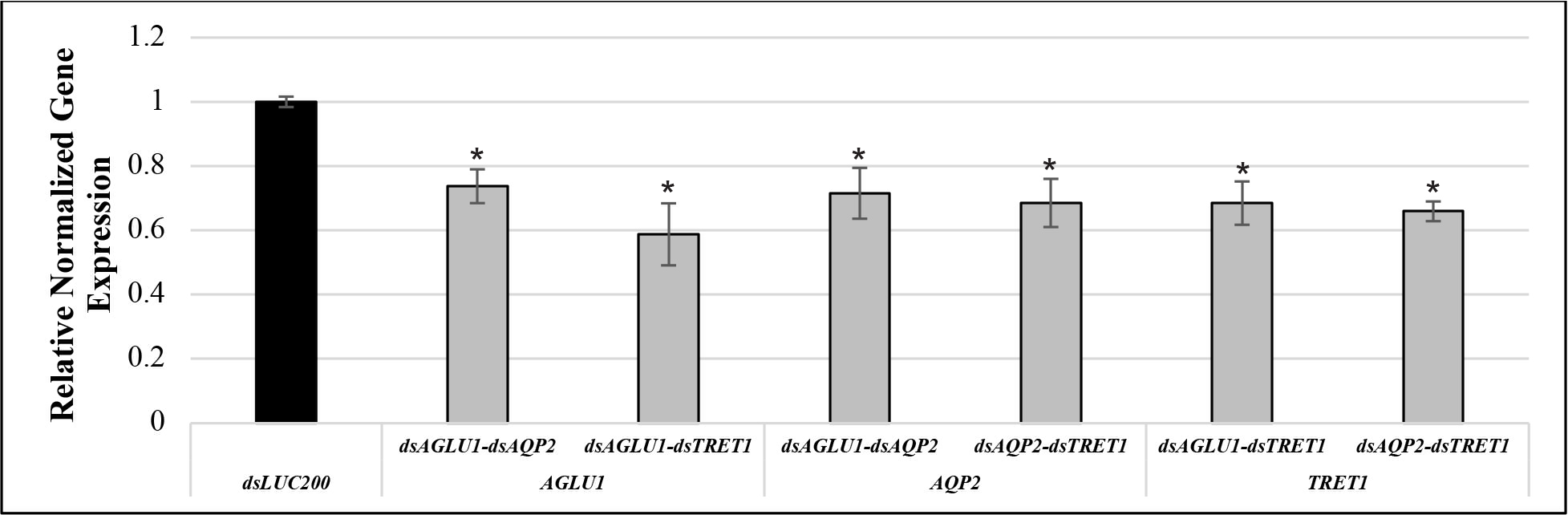

Supplement: Supplementary file 6 [file Image_4.tif]

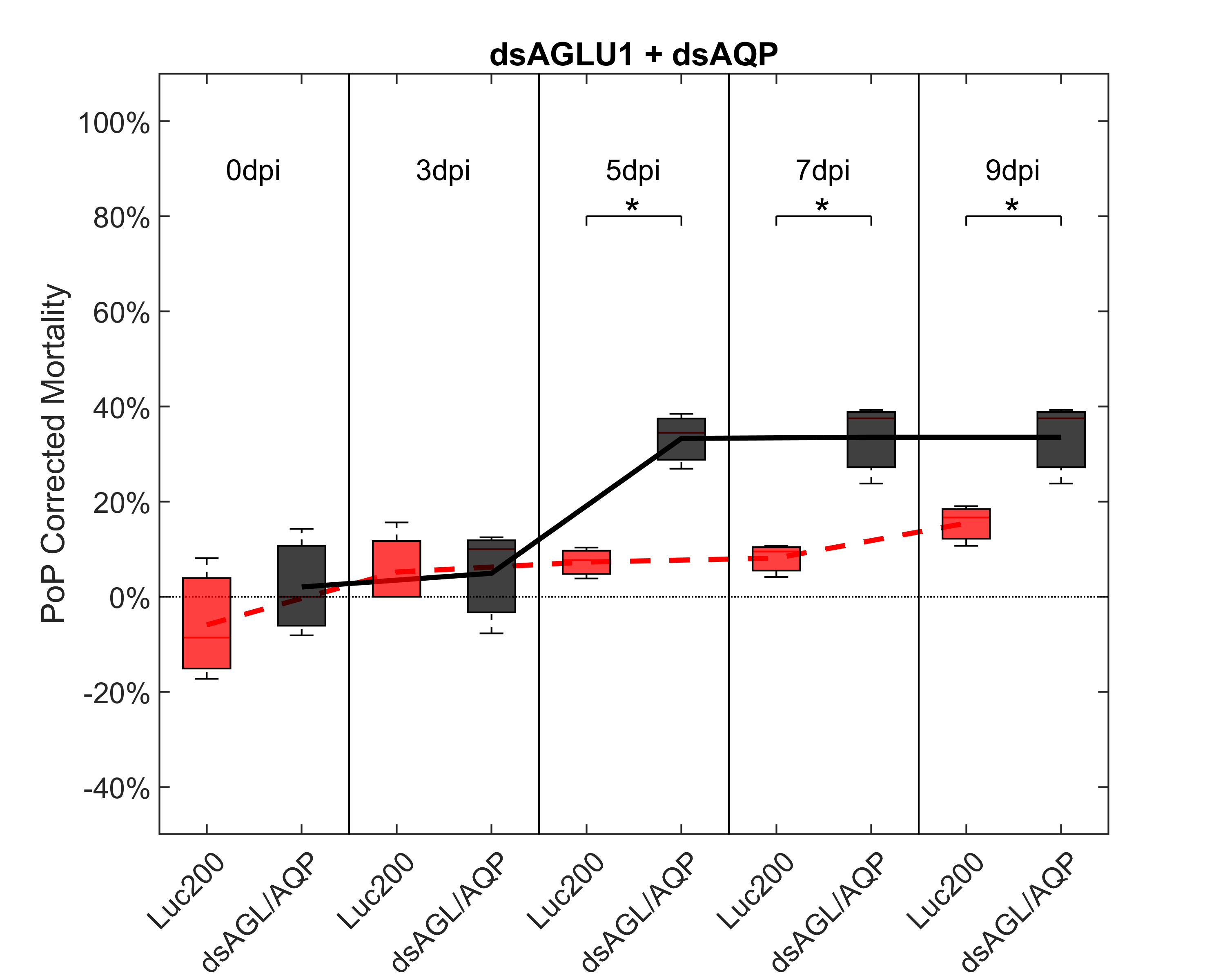

Supplement: Supplementary file 7 [file Image_5.tif]

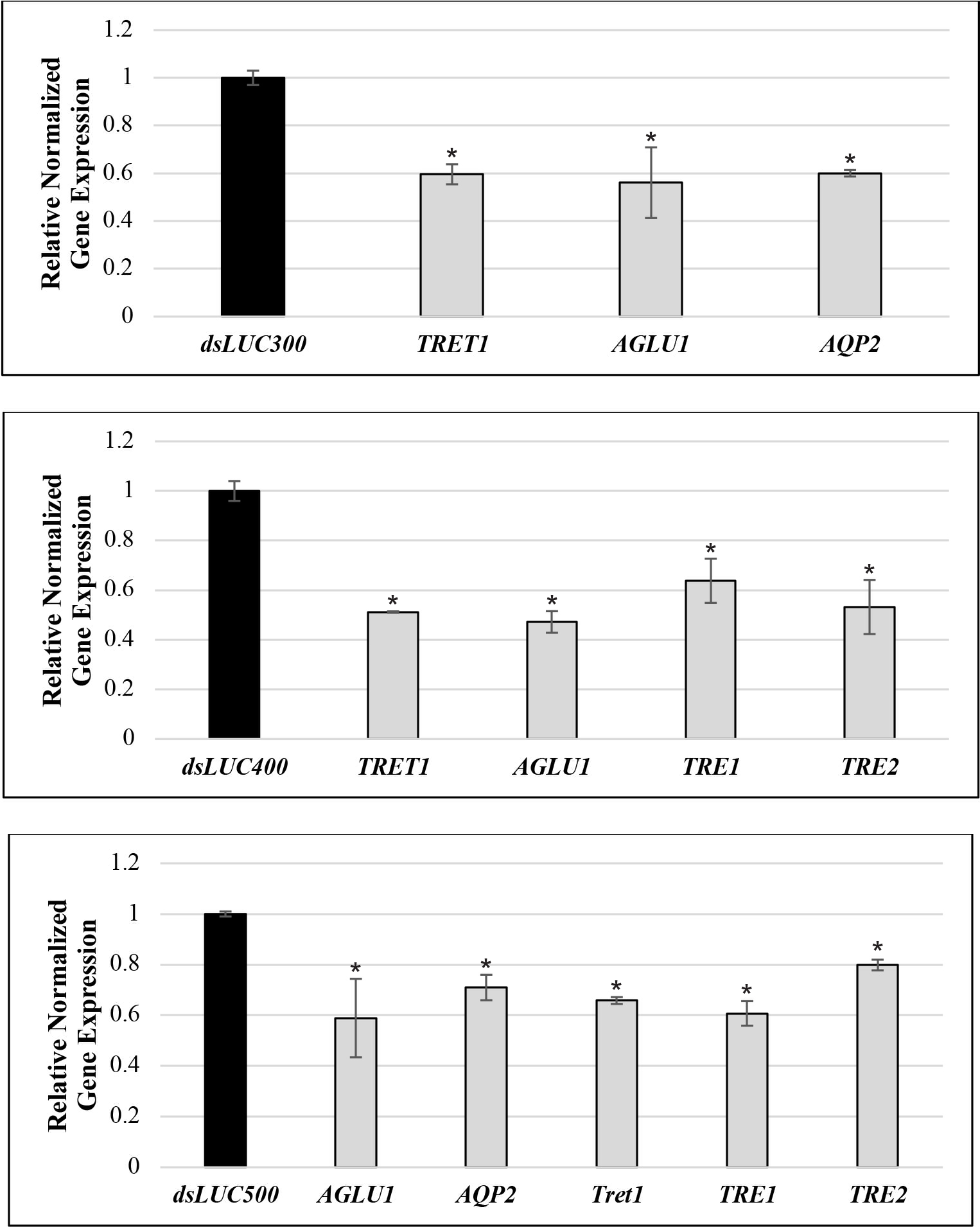

Supplement: Supplementary file 8 [file Image_6.tif]
